# Mahogunin Ring Finger 1 regulates pigmentation by controlling the pH of melanosomes in melanocytes and melanoma cells

**DOI:** 10.1007/s00018-021-04053-9

**Published:** 2021-12-18

**Authors:** Julia Sirés-Campos, Ana Lambertos, Cédric Delevoye, Graça Raposo, Dorothy C. Bennett, Elena Sviderskaya, Celia Jiménez-Cervantes, Conchi Olivares, José Carlos García-Borrón

**Affiliations:** 1 University of Murcia, Murcia, Spain; 2 Present Address: Institut Curie, UMR144, Structure and Membrane Compartments, PSL Research University, CNRS, 75005 Paris, France; 3 Institut Curie, UMR144, Cell and Tissue Imaging Facility (PICT-IBiSA), PSL Research University, CNRS, 75005 Paris, France; 4 Molecular and Clinical Sciences Research Institute, St. George’s, University of London, London, SW17 0RE UK

**Keywords:** Mahogunin Ring Finger 1 (MGRN1), Melanin, Tyrosinase, Lysosome-related organelles, Melanosomal pH, Mucolipin 3 (MCOLN3)

## Abstract

Mahogunin Ring Finger 1 (MGRN1) is an E3-ubiquitin ligase absent in dark-furred *mahoganoid* mice. We investigated the mechanisms of hyperpigmentation in *Mgrn1*-null melan-md1 melanocytes, *Mgrn1*-KO cells obtained by CRISPR-Cas9-mediated knockdown of *Mgrn1* in melan-a6 melanocytes, and melan-a6 cells depleted of MGRN1 by siRNA treatment. *Mgrn1*-deficient melanocytes showed higher melanin content associated with increased melanosome abundance and higher fraction of melanosomes in highly melanized maturation stages III–IV. Expression, post-translational processing and enzymatic activity of the rate-limiting melanogenic enzyme tyrosinase measured in cell-free extracts were comparable in control and MGRN1-depleted cells. However, tyrosinase activity measured in situ in live cells and expression of genes associated with regulation of pH increased upon MGRN1 repression. Using pH-sensitive fluorescent probes, we found that downregulation of MGRN1 expression in melanocytes and melanoma cells increased the pH of acidic organelles, including melanosomes, strongly suggesting a previously unknown role of MGRN1 in the regulation of melanosomal pH. Among the pH regulatory genes upregulated by *Mgrn1* knockdown, we identified those encoding several subunits of the vacuolar adenosine triphosphatase V-ATPase (mostly *Atp6v0d2*) and a calcium channel of the transient receptor potential channel family, Mucolipin 3 (*Mcoln3*). Manipulation of expression of the *Mcoln3* gene showed that overexpression of *Mcoln3* played a significant role in neutralization of the pH of acidic organelles and activation of tyrosinase in MGRN1-depleted cells. Therefore, lack of MGRN1 led to cell-autonomous stimulation of pigment production in melanocytes mostly by increasing tyrosinase specific activity through neutralization of the melanosomal pH in a MCOLN3-dependent manner.

**Supplementary Information:**

The online version contains supplementary material available at 10.1007/s00018-021-04053-9.

## Introduction

The skin and hair color of mammals is largely determined by the amount and relative proportions of two types of pigment derived from the amino acid l-tyrosine, eumelanins and pheomelanins [1]. The synthesis of both types of melanin pigment takes place in melanocytes, within specialized lysosome-related organelles (LROs) called melanosomes [2, 3]. Melanogenically active melanosomes are derived from early endosomes that receive various components required for their pigmentation, like a set of melanogenic enzymes transported to the melanosome precursors through the biosynthetic-secretory and endosomal pathways [2, 4]. These enzymes are the rate-limiting tyrosinase (TYR), a copper-containing bifunctional enzyme catalyzing two consecutive reactions, hydroxylation of l-tyrosine to l-DOPA and oxidation of l-DOPA to DOPAquinone [5], and two similar metalloproteins, the tyrosinase-related proteins 1 and 2 (TYRP1 and TYRP2/DCT) that catalyze subsequent reactions in the eumelanogenic pathway [6–8]. Melanosomes undergo a complex multistage maturation. In their first stage (stage I melanosomes or premelanosomes), they display all the features of early endosome, where intraluminal fibrils composed of the melanosomal glycoprotein PMEL are progressively formed. The formation of physiological amyloid fibrils is completed in stage II melanosomes. Melanin synthesized by TYR and TYRP1/2 is deposited on these fibrillar sheets, leading to stage III pigmented melanosomes. In highly pigmented stage IV melanosomes, the intraluminal structure is no longer visible due to the extensive accumulation of pigment and the melanogenic activity is reduced or absent [9].

Hundreds of genes contribute to the regulation of mammalian skin pigmentation, including genes involved in developmental processes, melanocyte proliferation, survival and intracellular trafficking, melanosome biogenesis, movement and transfer to keratinocytes, homeostasis of the melanosomal milieu, melanin synthesis and others [10, 11]. Many of these genes were identified by virtue of coat color changes in mutant mice. One of them, *Mgrn1*, was identified by positional cloning of the *mahoganoid* mouse mutation [12] causing fur darkening, mitochondrial dysfunction, congenital heart defects and spongiform neurodegeneration in adult mice [13, 14]. *Mgrn1* encodes Mahogunin Ring Finger 1 (MGRN1), a RING-finger nuclear-cytoplasmic E3-ubiquitin ligase ubiquitously expressed in mammals [12, 15, 16] and found in most human tissues (proteinatlas.org/ENSG00000102858-MGRN1/tissue). MGRN1 displays ubiquitin ligase activity towards a number of substrates including TSG101 [17–21], a component of the endosomal sorting complex required for transport-1 (ESCRT1) involved in trafficking of TYRP1 to melanosomes [22], mitofusin1 and GP78 involved in the control of mitochondrial dynamics [23–26] and α-tubulin [26, 27]. MGRN1 may also participate in clearance of misfolded proteins and polyglutamine proteins such as huntingtin and ataxin-3, to prevent protein aggregation and toxicity [28–30]. Moreover, MGRN1 interacts with the melanocortin 1 receptor (MC1R) and inhibits its functional coupling to the cAMP cascade upon binding of agonist melanocortin peptides, but it does not appear to ubiquitylate the receptor [31]. Since activation of cAMP signaling downstream of MC1R is required for stimulation of TYR activity and for the switch from basal pheomelanin production to eumelanin synthesis, MGRN1-mediated inhibition of MC1R signaling could potentially account for the modulation of pigmentation by MGRN1. However, hyperpigmentation in *Mgrn1-null mahoganoid* melanocytes appears as a cell-autonomous process independent of MC1R stimulation by exogenous melanocortins [32, 33]. Thus, the molecular basis of the association of hyperpigmentation and MGRN1 loss remains unclear.

The aim of this study was to analyze the molecular mechanisms accounting for increased melanogenesis in *Mgrn1*-null melanocytes. We show that stimulation of melanogenesis is independent of induction of TYR expression and is due to the neutralization of melanosomal pH, mostly associated with induction of the Ca^2+^ transporter MCOLN3. Moreover, we show that repression of MGRN1 in heterologous HEK293 cells also results in neutralization of intracellular acidic vesicles, thus suggesting that MGRN1 might be a new general regulator of the luminal pH of lysosomal lineage organelles, including LROs.

## Materials and methods

### Reagents

General laboratory reagents, forskolin and acridine orange were from Sigma-Aldrich (St. Louis, MO), Calbiochem (Darmstadt, Germany), Merck (Darmstadt, Germany) or Prolabo (Barcelona, Spain), unless specified otherwise. Lipofectamine 2000 was from Invitrogen (Carlsbad, CA). Reagents for electrophoresis and Western blotting were from Bio-Rad (Richmond, CA). Radioactive reagents were from National Diagnostics (Nottingham, UK), Fluka (Buchs, Switzerland) and PerkinElmer (Boston, MA). The MCOLN3 agonist SN2 was from Tocris Bioscience (Abingdon, OX) and DAMP-DNP and a polyclonal anti-DNP (Acidic Granule Kit) from Oxford Biomedical Research (Rochester Hills, MI).

### Cell culture

Cell culture reagents were from Gibco (Gaithersburg, MD). MNT-1 melanocytes were cultured in DMEM supplemented with 20% fetal bovine serum (FBS), 10% AIM-V medium, 1% nonessential amino acids and 1% sodium pyruvate. Mouse melan-md1 and melan-a6 cells were grown in RPMI 1640 medium with 10% FBS, 200 nM 12-*O*-tetradecanoylphorbol-13-acetate (TPA), 100 U/ml penicillin and 100 μg/ml streptomycin sulfate. Mouse melanoma B16F10 cells were a kind gift from Prof. JN Rodríguez-López (University of Murcia, Spain). HEK293T and B16F10 cells were grown in DMEM, 10% FBS, 100 U/ml penicillin and 100 μg/ml streptomycin sulfate. Usually, cells were cultured for a maximum of 5 passages.

### Expression constructs and transfection

*TYR* (Uniprot no. P14679) and HA epitope-tagged human *MGRN1*, isoform S(+) (UniProt no. O60291-1) were cloned into the pcDNA3 expression vector (Invitrogen, Carlsbad, CA) as previously described [8, 31]. Other expression constructs such as MCOLN3-GFP (Uniprot no. Q8TDD5) were purchased from Origene (Rockville, MD). Constructs were verified by automated sequencing. Cells were transfected using Lipofectamine 2000 according to manufacturer’s instructions.

### RNAi transfection and generation of CRISPR/Cas9-based Mgrn1-KO cells

Mouse or human melanocytic cells (2 × 10^5^) were seeded in a 6-well plate and transfected the next day with *MGRN1* or *Mgrn1*-directed siRNA oligonucleotides from Horizon (PerkinElmer), or *MCOLN3-, Mcoln3*- or *Atp6v0d2*-specific siRNA oligonucleotides from Origene, as described [33]. Non-targeting siRNA was used as control. Cells were used 48 h after transfection. CRISPR/Cas9-based *Mgrn1*-KO mouse melanocytes or melanoma cells were obtained as described elsewhere [33]. Oligonucleotide sequences are available upon request.

### Staining of acidic organelles

For DAMP staining, cells were serum-starved 30 min, incubated 30 min with 30 μM DAMP-DNP at 37 °C [34], then washed with cold medium before fixation. For labeling with acridine orange (AO), cells grown in fluorodishes were incubated 20 min with 5 μg/ml AO at 37 °C [35], then washed with PBS1x at 37 °C before acquisition of the images.

### Enzyme activity and melanin content determinations

The DOPA oxidase (DO) activity of TYR was determined using cell-free, detergent-solubilized extracts. Cells were solubilized in 1% Igepal, 1% phenylmethylsulfonyl fluoride, 50 mM phosphate buffer pH 6.8 and centrifuged at 2000 rpm for 5 min. DO activity was measured spectrophotometrically according to Winder and Harris [36] with minor modifications. Briefly, cell extracts (usually 50–100 µg protein/assay) were incubated in the presence of 4 mM 3-methyl-2-benzothiazolinone (MBTH) and different concentrations of l-DOPA (0.1 to 2.5 mM) in 50 mM phosphate buffer pH 6.8 in a 96-well plate. Absorbance at 490 nm was measured in a microplate reader every 10 min for 1 h and enzymatic activity was expressed as the slopes of the corresponding graphs, calculated with the GraphPad Prism Software (San Diego, CA). For the analysis of kinetic parameters, a Michaelis–Menten plot was obtained using GraphPad Prism. The tyrosine hydroxylase (TH) activity in cell-free extracts was determined by a radiometric method [37]. One unit was defined as the amount of enzyme catalyzing the hydroxylation of 1 μmol of l-tyrosine/min, in the presence of a 50 μM concentration of the substrate and 10 μM DOPA as cofactor. A modification of the protocol was designed to measure TH activity in live cells. In this case, all manipulations including cell culture were carried out in a containment facility of the Radioprotection Service of the University of Murcia and care was taken to avoid exposure to tritiated water vapor. Briefly, 0.022 μM l-[3,5-^3^H]-tyrosine (1 μCi/mmol) was added to the medium and cells were incubated overnight at 37 °C. The medium was recovered and treated as described for the reaction mix in the in vitro assay. Briefly, a Celite 545:activated carbon mixture (1:1) was added to 500 μl of medium, vortexed for 10 min and centrifuged at 12,000 rpm during 5 min at 4 °C. 100 μl of the supernatants was mixed with 2 ml of scintillation cocktail and analyzed in a scintillation counter (Perkin Elmer TriCarb 2900). The cpm obtained were normalized by time and cell number.

Ornithine decarboxylase (ODC) activity was determined by measuring ^14^CO_2_ released from 30 μM l-[1-^14^C] ornithine. Cells were lysed in solubilization buffer (50 mM Tris–HCl, 1% Igepal and 1 mM EDTA) and centrifuged at 14,000×*g* for 20 min. A fraction of the supernatant was taken to a final volume of 50 µl with buffer A (10 mM Tris–HCl, 0.25 M sucrose, 0.1 mM pyridoxal phosphate, 0.2 mM EDTA and 1 mM dithiothreitol). The reaction was performed in glass tubes tightly closed with rubber stoppers equipped with two hanging disks of filter paper wetted in 0.5 M benzethonium hydroxide dissolved in methanol. The samples were incubated at 37 °C for 1 h, and the reaction was stopped by adding 0.5 ml of 2 M citric acid. The filter paper disks were transferred to scintillation vials and counted by liquid scintillation [38].

Melanin content was determined after alkaline solubilization using a colorimetric assay based on determination of the absorbance at 405 nm after digestion with KOH 0.85 M during 4 h at 100 °C [39].

### Tyrosine uptake

Cells were treated with 1 mM phenyl thiourea (PTU), a TYR inhibitor and the culture medium was enriched with 100 μM l-[3,5-^3^H]- tyrosine (1 μCi/mmol) during 12 h. Cultures were PBS-washed twice and radioactivity was measured in the cellular pellet in a scintillation counter.

### Polyamine analysis

Cells were homogenized in 0.4 M perchloric acid and centrifuged at 10000xg for 15 min. Polyamines in the supernatant were dansylated according to a standard method [40]. Dansylated polyamines were separated by HPLC, using a BondaPak C18 column from Waters and an acetonitrile/water gradient (70:30–96:4 during 30 min) as mobile phase, at a flow rate of 1 ml/min. We used 1,6-hexanediamine and 1,7-heptanediamine as internal standards, and standard solutions of putrescine, spermidine, spermine and isopentylamine for column calibration. Detection of the derivatives was achieved with a Waters 420-AC fluorescence detector (Millipore), with excitation and emission at 340 and 435 nm, respectively.

### Endoglycosidase treatment

For deglycosylation studies, cell extracts were incubated at 37 °C for 4 h with 5 U of either endoglycosidase H or peptide-*N*-glycosidase F (PNGase F) in 50 mM phosphate buffer, pH 7.0, containing 10 mM EDTA and 0.1% SDS. Samples for PNGase F digestion were heated at 95 °C for 5 min prior to incubation at 37 °C.

### Immunoblotting

Western blotting was performed as described [31, 41] using the following antibodies: αPEP1, αPEP7, αPEP8 and αPEP13 (a kind gift from Dr V. Hearing (National Institutes of Health, Bethesda, MD, U.S.A.), αERK1/2 from Santa Cruz (Dallas, TX), αFLAG, αHA and αGAPDH from Sigma-Aldrich, αMGRN1 and αMCOLN3 from Origen and αATP6V0D2 from Aviva System (San Diego, CA).

### Confocal fluorescence microscopy imaging

Cells grown on coverslips were fixed for 15 min at room temperature with 4% formaldehyde, then permeabilized with 0.5% Triton X-100 (v/v) and blocked with 5% BSA before staining. Coverslips were analyzed on a SP8 Leica laser scanning confocal microscope (Leica Microsystems GmbH; Wetzlar, Germany). Backscattered light (reflectance) was collected to image the matrix surrounding cells. In addition to the primary antibodies described above, we used αDNP (Oxford Biomedical Research), αLAMP1 and αNKI (Thermo Fisher Scientific; Waltham, MA), and αHMB45 and αTYRP1 (Abcam; Cambridge, UK) at a 1:200 dilution. Secondary antibodies (Alexa 488, 568 or 647, used at 1:200) were from Thermo Fisher Scientific.

### Pearson’s correlation coefficient

Pearson’s correlation coefficient between two channels was quantified using JACoP plugin of ImageJ from one frame of the live-cell acquisition. For negative control, similar measurements were performed after rotating 30° the GFP channel on the same images.

### Electron microscopy

Cells cultured on coverslips and transfected with control and *Mgrn1* siRNAs were fixed in 2.5% (v/v) glutaraldehyde, 0.1 M cacodylate buffer for 24 h, post-fixed with 1% (w/v) osmium tetroxide supplemented with 1.5% (w/v) potassium ferrocyanide, dehydrated in ethanol and embedded in Epon [42]. Ultrathin sections were prepared with a Reichert UltracutS ultramicrotome (Leica Microsystems) and contrasted with uranyl acetate and lead citrate. Electron micrographs were acquired on a Tecnai Spirit Electron Microscope (Thermo Fisher Scientific) equipped with a 4 k CCD camera (EMSIS GmbH, Muenster, Germany) using ITEM software (EMSIS). The number of melanosomes per cell in each maturation stage was quantified manually.

### Microarray analysis

Total RNA from cultured melan-md1 and melan-a6 cells was extracted with RNeasy (QIAGEN, Hilden, Germany) (*n* = 4 biological replicas for each cell type). cDNA was synthesized using Super-Script^®^ III, amplified and processed following Affymetrix microarray instructions. Samples were hybridized using suitable mouse gene 2.0 chip microarray matrixes. Differential gene expression was analyzed using the GSEA platform. Data sets containing 41,345 native features were collapsed into gene symbols and the resulting 16,705 genes were ranked by expression fold change. A heatmap showing the 50 most upregulated and downregulated genes in melan-md1 cells compared with melan-a6 cells was built by means of the GSEA platform.

### Gene expression analysis by real-time PCR

Cells were serum-deprived at least 12 h before RNA extraction with RNeasy. One microgram of RNA was reverse-transcribed using Super-Script^®^ III. RT-PCR was performed using the Power SYBR Green PCR Master Mix (Applied Biosystem, Thermo Fisher Scientific) on an ABI 7500 Fast Real Time PCR System. The primer sequences are available upon request.

### Statistical analysis

Experiments were performed with at least three biological replicates. No samples were excluded from any analyses. Subpopulations of cells were randomly assigned to treatments. Blind analysis was not performed in this study. Statistical significance was assessed using GraphPad Software. Data met the assumptions of the test used. We used D’Agostino–Pearson omnibus normality test for Gaussian distribution. Unpaired two-tailed Student’s *t* test and one-way ANOVA with Tukey post-test for multiple comparisons were performed when variance among groups was not statistically different. Unless otherwise specified, results are expressed as mean ± SEM; p values were calculated using two-sided tests. *p* values of less than 0.05 were considered significant. * *p* < 0.05, ** *p* < 0.01, *** *p* < 0.001, and **** *p* < 0.0001.

## Results

### Increased melanogenic activity, but not tyrosinase abundance, in MGRN1-depleted melanocytes

**Fig. 1:**
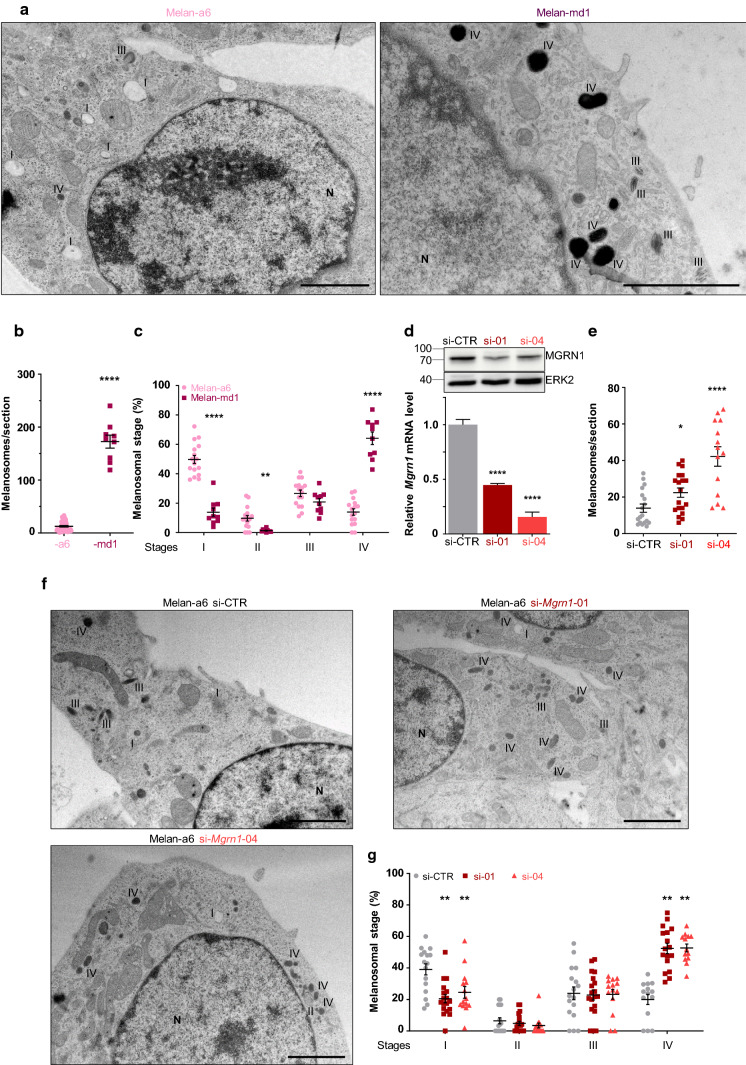
Ultrastructural analysis of MGRN1-deprived mouse melanocytes. **a** Electron microscopy analysis of ultrathin sections of epon embedded control melan-a6 melanocytes and *Mgrn1*-null melan-md1 cells. **b** Quantification of total number of melanosomes on EM sections (*n* ≥ 10). **c** Distribution of melanosomes in the four maturation stages. The melanosomes in at least 10 independent sections were classified in stages I–IV according to their morphology and melanin content, as shown in panel **a**. Stage III and IV melanosomes were distinguished by the lower degree of melanization and the occurrence of identifiable fibrils not completely obliterated by melanin deposits in stage III melanosomes as opposed to the homogeneously dense appearance of stage IV organelles. The graph shows the percentage of organelles in each stage relative to the total number of melanosomes. **d** Depletion of MGRN1 in melan-a6 cells using two individual siRNAs (si-01 and si-04). The upper Western blot shows the abundance of the MGRN1 protein in cells treated with a control siRNA (siCTR) or with *Mgrn1*-specific siRNAs, with ERK2 as a control for comparable loading. The lower bar graph depicts the relative expression of *Mgrn1* mRNA in melan-a6 cells treated with control and *Mgrn1*-directed siRNA, estimated by qPCR (*n* = 6). **e** Quantification of total number of melanosomes on EM sections of melan-a6 cells treated with control or *Mgrn1*-directed siRNA as in panel **d** (*n* ≥ 14). **f** 60-nm electron micrographs of melan-a6 cells treated with control siRNA (siCTR) or with two different *Mgrn1*-directed siRNAs. **g** Distribution of melanosomes in the four maturation stages in siCTR or *Mgrn1*-siRNA-treated melan-a6 cells (*n* ≥ 14), estimated as in panel **c**. *N* nucleus. Scale bar: 2 µm

We previously reported that *Mgrn1*-KO cells derived from either melan-a6 mouse melanocytes or B16 mouse melanoma cells are darker and show a higher melanin content than control, MGRN1-expressing cells [33]. Moreover, *Mgrn1*-null melan-md1 mouse melanocytes derived from *Mgrn1*^*md−nc*^*/Mgrn1*^*md−nc*^ mice are also more pigmented in vitro than control melan-a6 *Mgrn1*^+*/*+^ cells [32, 33]. To further investigate the cellular basis of such hyperpigmentation, we performed a detailed study at the ultrastructural level allowing to resolve qualitatively and quantitatively alterations of subcellular and organellar organization and morphology. Analysis by conventional electron microscopy showed that *Mgrn1*-null melan-md1 cells harbored more melanosomes than Mgrn1-expressing melan-a6 cells (Fig. 1a and b). Within melan-md1 cells, the percentage of highly melanized stage IV melanosomes was also higher, since mature melanosomes accounted for more than 50% of the total melanosome population, whereas lower amounts of stages I and II were observed. Conversely, more than 50% of melanosomes in melan-a6 cells were stages I and II (Fig. 1a and c). However, these results must be interpreted with caution because of senescence-like features in the melan-md1 cell cultures used in this study and the less dramatic hyperpigmentation phenotype of *Mgrn1*-KO melan-a6 cells completely lacking MGRN1 [33]. Accordingly, we performed a similar ultrastructural analysis using melan-a6 mouse melanocytes treated with two different *Mgrn1*-directed siRNAs (Fig. 1d–g). For both siRNAs, efficient repression of *Mgrn1* was verified by real-time PCR and by Western blot (Fig. 1d) and was shown to significantly increase the number of melanosomes in melan-a6 melanocytes (Fig. 1e). Moreover, the fraction of stage I melanosomes was lower in MGRN1-depleted cells whereas the one of highly melanized stage IV melanosomes was significantly higher (Fig. 1f, g). We obtained consistent results in MNT-1 human melanoma cells, characterized by melanosome biogenesis [34] and protein or gene expression profiles [43] comparable to normal human melanocytes, indicating that the pigmentation stimulatory effect of MGRN1 downregulation was not restricted to mouse cells (Supplementary Fig. S1). Overall, these data showed that (i) MGRN1-deprived melanocytes and melanoma cells were more pigmented than their corresponding controls, (ii) this stimulation of pigmentation was cell-autonomous as it occurred in the absence of keratinocyte-secreted factors or exogenous MC1R agonists and (iii) hyperpigmentation likely resulted from both an increased number of melanosomes and a higher percentage of melanosomes in highly pigmented stages III and IV, indicative of a higher rate of melanogenesis.

**Fig. 2:**
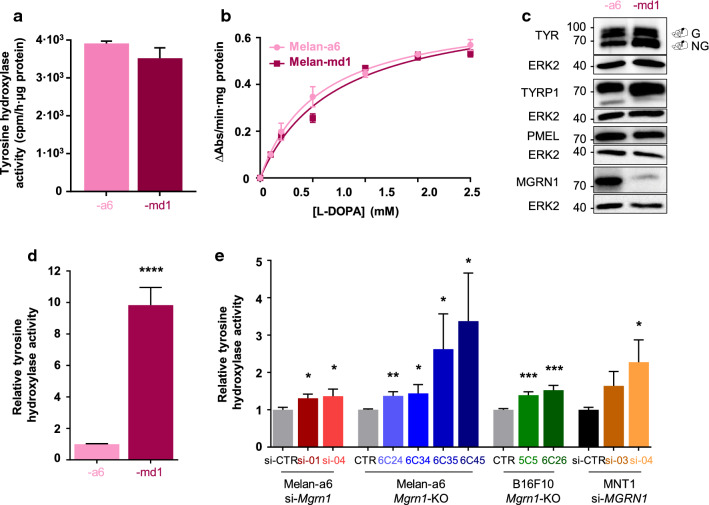
Tyrosinase activity in control and Mgrn1-depleted cells. **a** Tyrosine hydroxylase activity in cell-free extracts from mouse melan-a6 and -md1 melanocytes (*n* ≥ 8). Data were expressed as cpm/h/μg protein. **b** Michaelis–Menten plot for DOPA oxidase activity in cell-free extracts from melan-a6 and -md1 cells (*n* = 3). **c** Representative immunoblots of melanosomal proteins (TYR, TYRP1, PMEL17 and MGRN1 in melan-a6 and -md1 melanocytes. ERK2 was used as loading control. **d** Tyrosine hydroxylase activity measured in live cells and normalized to total protein in melan-a6 and -md1 melanocytes (*n* ≥ 8). **e** Changes in tyrosine hydroxylase activity in live cells upon abrogation of Mgrn1 expression by CRISPR-Cas9 (*Mgrn1*-KO melan-a6 cells, *n* = 8 and B16F10 mouse melanoma cells, *n* = 10), or upon downregulation of MGRN1 in melan-a6 and MNT-1 cells by treatment with two different specific siRNAs (*n* ≥ 4). Data are expressed as relative activity respect to the corresponding controls.

Since the rate-limiting melanogenic enzyme is TYR, we compared its activity in melanocytes expressing normal or reduced levels of MGRN1. Surprisingly, we found a comparable rate of tyrosine hydroxylation in assays performed with cell-free detergent-solubilized extracts of melan-a6 and melan-md1 cells (Fig. 2a). To strengthen this unexpected result, we measured the DOPA oxidase activity in cell-free detergent-solubilized extracts, at different substrate concentrations allowing for calculation of kinetic parameters. Again, similar reaction velocities and comparable Km values (0.56 ± 0.05 mM for melan-a6 and 0.73 ± 0.10 mM for melan-md1) were obtained (Fig. 2b). In agreement with these results, the abundance of TYR and the melanosomal proteins PMEL and TYRP1 was also comparable according to Western blot analysis (Fig. 2c). It is known that TYR levels are partially regulated by proteasomal degradation [44–48]. However, the comparable abundance of TYR and the similar enzymatic activity in cell-free extracts excluded the simple possibility that impaired proteasomal degradation of the enzyme due to absence of MGRN1 might account for hyperpigmentation in MGRN1-null cells.

In sharp contrast with the similar TYR activities measured in cell-free extracts, the tyrosine hydroxylase activity measured in live cells (i.e., in situ) was roughly tenfold higher in melan-md1 cells compared with melan-a6 cells (Fig. 2d). We extended these analyses to mouse *Mgrn1*-KO cells generated by CRISPR-Cas9 and to mouse and human melanocytic cells depleted of MGRN1 following treatment with various siRNAs (Fig. 2e). For all these cell types, we detected significant increases in the rate-limiting tyrosine hydroxylase activity measured in situ. Therefore, TYR-specific activity increased upon repression of MGRN1 in the absence of comparable changes in its level of expression.

### Neutralization of the melanosomal pH in MGRN1-deprived melanocytes and melanoma cells

We investigated several possible mechanisms compatible with the higher TYR-specific activity within live MGRN1-deficient cells. TYR and other melanosomal proteins undergo a complex post-translational processing during their traffic from the ER, Golgi and further to melanosomes. This maturation is important for the acquisition of the catalytically active conformation and involves N-glycosylation in several glycosylation sites. Since inefficient processing of the enzyme most often results in aberrant glycosylation with retention within the ER, we compared the glycosylation pattern of TYR in melan-a6 and melan-md1 melanocytes by means of Endo H or PNGase glycosidase digestion followed by Western blot. Endo H digests high mannose glycans found in incompletely processed glycoproteins [49]. Thus, sensitivity to Endo H allows distinguishing incompletely processed, Endo H-sensitive TYR from the mature, Endo H-resistant enzyme [50–53]. Conversely, PNGase F removes all glycans, irrespective of their degree of maturation. The digestion patterns of TYR from melan-a6 and melan-md1 cells were comparable, suggesting that there was no significant change in the rate of TYR post-translational processing in MGRN1-null cells (Supplementary Fig. S2a).

On the other hand, in the in vivo tyrosine hydroxylase assay the radioactive substrate is added to the culture medium, and therefore, the measured TYR activity might be affected by changes in tyrosine uptake from the medium, i.e., by changes in substrate availability. Accordingly, we compared the rate of uptake of tritiated tyrosine by wild-type or *Mgrn1*-null cells. Cells were incubated with the radioactive tracer in the presence or absence of the potent TYR inhibitor phenylthiourea (PTU), washed and counted for their tritium content. PTU was used because radiolabeled tyrosine is tritiated in positions 3 and 5 of the indole ring and one of the tritium atoms is removed upon catalytic conversion into l-DOPA. Therefore, differences in TYR activity would interfere with the assay by leading to different rates of tritium release from radiolabeled tyrosine in both cell types. The amount of incorporated tyrosine was lower for MGRN1-deficient cells, both in the presence or absence of PTU (Supplementary Fig. S2b), thus ruling out increased substrate uptake as a cause of high TYR-specific activity in live MGRN1-null cells.

It has been reported that the melanosomal pH has a major effect on TYR activity. Therefore, we hypothesized that lack of MGRN1 might increase the melanosomal pH to activate the enzyme. To test this hypothesis, we first verified the strong pH dependence of the rate-limiting tyrosine hydroxylase activity of TYR by measuring the activity of crude detergent-solubilized extracts in phosphate buffer at different pH values (Supplementary Fig. S2c). We found that increasing the pH of the reaction medium from 5 (an acidic pH value typical of late endosomes and lysosomes [54] to a near-neutral value of 6.8 augmented about sixfold the specific activity of TYR measured in vitro. Moreover, activation of TYR upon neutralization of the reaction medium was very similar for melan-a6 and melan-md1 extracts. Treatment of melan-a6 cells with the lysosomotropic alkalinizing agent NH_4_Cl markedly stimulated TYR activity measured in live cells, but had a minor, non-significant effect on melan-md1 melanocytes (Supplementary Fig. S2d). This treatment also increased significantly pigment production in melan-a6 cells but not in melan-md1 melanocytes, as shown by visual inspection of cell pellets and by colorimetric determination of melanin contents (Supplementary Figs S2e, f). Overall, these data suggested that a less acidic pH in the melanosomes of *Mgrn1*-null cells compared with *Mgrn1*-expressing melanocytes might be a major contributor to the higher TYR-specific activity of the former. Since stage II melanosomes contain TYR in an inactive state partly because of the low pH of these organelles [55], a less acidic pH in *Mgrn1*-null cells might also increase the rate of melanosome maturation, and hence the percentage of mature melanosomes in these cells.

**Fig. 3:**
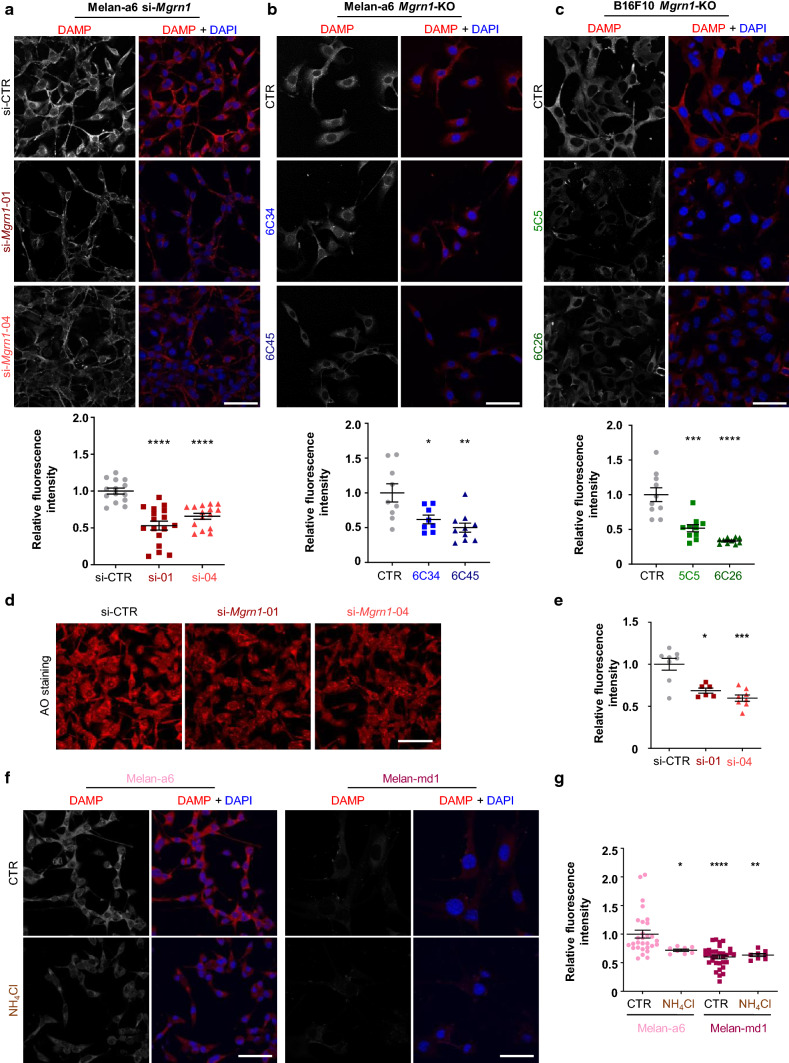
Analysis of the pH of acidic organelles using DAMP-DNP (DAMP) and Acridine Orange (AO). **a**–**c** Confocal micrographs of different cell types probed for 30 min with DAMP-DNP and stained for DNP (red) and nuclei (DAPI staining, blue). The histogram below each set of images corresponds to the quantification of the fluorescence intensity of DAMP-DNP staining, normalized to control. **a** Melan-a6 cells treated with control siRNA (siCTR) or *Mgrn1*-directed siRNA-01 or -04 (*n* ≥ 15). **b** Control (CTR) and CRISPR/Cas9 *Mgrn1*-KO clones of melan-a6 melanocytes (6C34 and 6C45 clones) (*n* ≥ 8). **c** CRISPR/Cas9 control (CTR) and *Mgrn1*-KO clones of B16F10 mouse melanoma cells (5C5 and 6C26 clones) (*n* ≥ 10). **d** Confocal microscopy images of AO-stained melan-a6 cells treated with control siRNA (siCTR) or *Mgrn1*-siRNA-01 or -04 before incubation with AO. **e** Quantification of the fluorescence intensity of AO-stained cells (*n* ≥ 6). Data are normalized by cell number in each image and referred to the control. **f** DAMP staining of control and NH_4_Cl-treated melan-a6 and melan-md1 melanocytes. Nuclei were stained with DAPI and are shown in the merged images. **g** Relative fluorescence intensity of DAMP staining in control and NH_4_Cl-treated cells (*n* ≥ 8, data normalized by cell number cell. Scale bar: 50 µm.

Therefore, we compared the pH of melanosomes in control and MGRN1-depleted cells by means of the acidotropic pH indicator *N*-{3-[(2,4-dinitrophenyl)amino]propyl}-*N*-(3-aminopropyl)methylamine dihydrochloride (DAMP). This probe accumulates in acidic organelles within live cells, where it can be detected with a specific anti-DNP primary antibody and Alexa-labeled secondary antibodies. The intensity of the resulting fluorescence of DAMP-labeled cells is inversely related to the pH of organelles such as melanosomes [56]. DAMP-associated fluorescence was lower in mouse melanocytes and melanoma cells when the expression of MGRN1 was abrogated by CRISPR-Cas9 or downregulated with specific siRNAs (Fig. 3a–c). Moreover, DAMP accumulation was also lower in melan-md1 melanocytes compared with genetically matched melan-a6 cells (Supplementary Fig. S3a, b). These data were consistent with an increased pH of LROs upon repression of MGRN1. An alternative possibility is that decreased fluorescence in Mgrn1-deprived cells might be due to higher absorbance of either excitation light or emitted fluorescence because of increased melanin content. This was unlikely because the observed trend was similar for cells with widely different melanin accumulation (melan-a6 derived CRISPR-Cas9 cultures or siRNA-treated cells are lightly melanized compared with our melan-md1 cultures). Nevertheless, we wanted to ascertain that the different intensity of DAMP labeling was actually due to a lower DAMP concentration in MGRN1-deprived cells, rather than being an artifact caused by their higher melanin content. To this end, we used two additional organelle labeling protocols with probes of different absorption/emission spectra. First, we visualized DAMP-labeled organelles in melan-a6 and melan-md1 cells with two secondary antibodies labeled with Alexa 647 or 488. We obtained comparable results, with a lower fluorescence emission in MGRN1-deprived cells, indicative of a higher luminal pH (Supplementary Fig. S3a, b). Next, we labeled control and MGRN1-depleted cells with acridine orange (AO), another acidophilic fluorescent probe that stains acidic organelles. siRNA-mediated repression of *Mgrn1* in melan-a6 cells, using the same oligonucleotides employed in Fig. 1e, resulted in lower AO labeling (Fig. 3d, e). A consistent decrease in AO labeling was obtained for melanocytes and melanoma cells lacking MGRN1 upon CRISPR-Cas9 knockdown (Supplementary Fig. 3c, d). Finally, incubation of melan-a6 cells with the neutralizing agent NH_4_Cl markedly decreased DAMP fluorescence to a level comparable with melan-md1 cells. Conversely, NH_4_Cl had a minor effect on the accumulation of DAMP in *Mgrn1*-null melan-md1 cells (Fig. 3f, g), in agreement with a higher organellar pH in these cells. Overall, these data strongly suggested that repression of MGRN1 caused a significant alkalinization of melanosomes, resulting in higher TYR-specific activity and melanin biosynthesis rate.

**Fig. 4:**
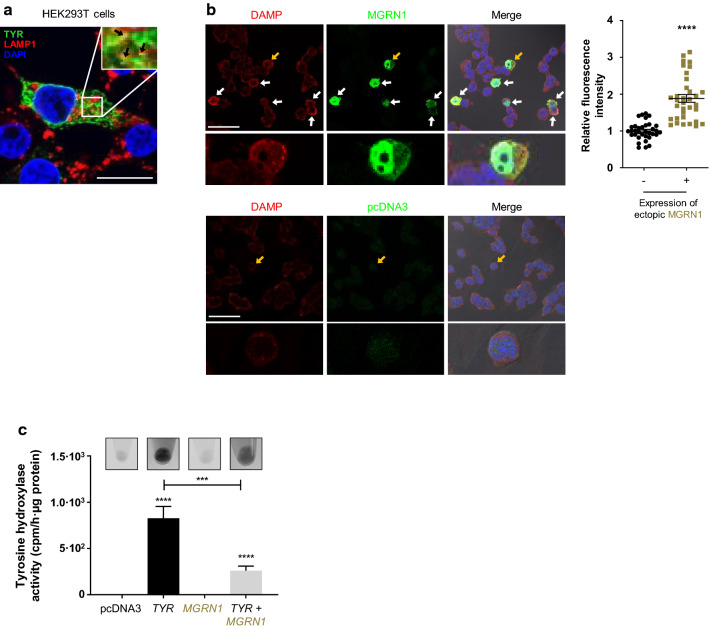
Changes in TYR activity ectopically expressed in HEK293T cells and in the pH of acidic organelles, upon forced expression of MGRN1. **a** Confocal microscopy images of HEK293T cells transfected to express TYR and immunostained for TYR (green) and endogenous LAMP1 (red). Black arrows in the 2.5 × magnified box show co-localization of both proteins (Pearson’s correlation coefficient: 58.3% ± 1.9). Scale bar: 20 µm. **b** Confocal images of HEK293T cells transfected with *Mgrn1*-*GFP* (upper rows) or with empty vector (lower rows) and stained for acidic organelles with DAMP (red). MGRN1 is shown in green. Arrows highlight MGRN1-expressing cells. Note that these cells displayed the highest intensity of DAMP staining. Scale bar: 50 µm. The enlarged images below each micrograph correspond to the cell indicated with a yellow arrow. The histogram shows the quantification of the relative fluorescence intensity of DAMP in cells expressing MGRN1, as compared with cells that do not express the exogenous protein at detectable levels (*n* = 34). **c** Tyrosine hydroxylase activity in live HEK293T cells transfected with empty vector (pcDNA3), or with TYR and MGRN1 expression constructs alone or in combination (*n* ≥ 8). Data were expressed as cpm/h/μg protein. Representative pictures of the corresponding cell pellets are shown above each bar of the graph.

We next wanted to find out whether the levels of MGRN1 expression could regulate the pH of acidic organelles in non-melanocytic cells or, conversely, if its effect on luminal pH was restricted to melanocytes. To this end, we co-transfected HEK293 cells with MGRN1 and TYR expression constructs. Early work by others showed that in non-melanocytic cells, ectopic melanosomal proteins of the TYR family are directed to lysosomes [57–59]. Therefore, this experimental setup should indicate whether lysosomes become more acidic upon forced expression of the S(+) MGRN1 isoform by means of two complementary readouts, namely DAMP labeling and TYR activity in vivo. Cells transfected to express TYR were immunostained for TYR and for the lysosomal marker LAMP1 and observed in a confocal microscope. Apposition of LAMP1 and TYR labeling was consistent with TYR expression in lysosomes (Fig. 4a). DAMP staining indicated a lower luminal pH in acidic compartments of MGRN1-overexpressing cells compared with untransfected cells (Fig. 4b). Of note, the intensity of DAMP-derived fluorescence in cells overexpressing or not MGRN1 was quantified in the same images to ensure identical conditions of image acquisition. Interestingly, DAMP and MGRN1 staining showed apposition in merged images, suggesting association of MGRN1 with acidic organelles. MGRN1 was also found in nuclei, in keeping with the presence of a canonical nuclear localization signal in the S(+) MGRN1 isoform [31]. Finally, increased TYR activity and higher deposits of melanin were found in cells transfected with TYR alone compared with cells coexpressing TYR and MGRN1 (Fig. 4c). The lower velocity of the rate-limiting melanogenic step and of pigment formation was fully consistent with a lower pH of the lumen of acidic organelles, including lysosomes, in MGRN1-overexpressing cells. In summary, the data presented thus far indicated that MGRN1 is a new regulator of the pH of melanosomes and other organelles of the lysosomal lineage, and that its expression correlates with lower intraorganellar pH. Moreover, the acidifying action of MGRN1 was not restricted to melanocytes.

### Role of the transient receptor potential cation channel mucolipin 3 (MCOLN3) in MGRN1-dependent regulation of the melanosomal pH

To study the molecular basis of the effect of MGRN1 on melanosomal pH, we looked at the differential expression of pH regulatory genes in *Mgrn1*-expressing versus *Mgrn1*-null cells. As a preliminary analysis, we compared the transcriptomic profiles of melan-a6 and melan-md1 cells. This was because the differences in intraorganellar pH and TYR activity were exacerbated in melan-md1 cells as compared with other *Mgrn1*-KO cells, which might increase the likelihood of identification of candidates. Total RNA from melan-a6 and melan-md1 cells was used to prepare cDNA, which was amplified and hybridized with an Affymetrix Mouse Gene 2.1 matrix. Interestingly, two of the most highly overexpressed genes in melan-md1 cells were *Atpv60d2*, encoding the d2 subunit of vacuolar-type V-ATPase and *Mcoln3*, coding for a cation channel involved in the regulation of the pH of endosomes and lysosomes, respectively [60] (Supplementary Fig. S4a). Moreover, melan-md1 cells showed aberrant expression of other genes potentially involved in the neutralization of melanosomal pH, notably those encoding for proteins of the polyamines biosynthesis pathway (not shown). The polyamines putrescine, spermidine and spermine are ubiquitous basic compounds with the potential to raise the pH of subcellular compartments. Moreover, polyamines have been shown to modulate melanogenesis although their effects on pigment production remain uncertain since both activation by putrescine [61] and by the putrescine synthesis inhibitor difluoromethylornithine (DFMO) [62] have been reported. We confirmed by qPCR the differential expression of genes encoding key enzymes and regulatory proteins of the polyamines biosynthetic pathway (Supplementary Fig. S4b). In *Mgrn1*-KO cells, we found consistent induction of expression of the genes encoding ornithine decarboxylase (ODC) and spermidine synthase (SPDST), which catalyze the formation of putrescine and spermidine, respectively. We also found induction of spermidine/spermine acetyl transferase (SSAT), an enzyme participating in the retroconversion of polyamines, as well as of the positive regulators of ODC activity AZIN1 and AZIN2. Conversely, expression of the ODC negative regulator AZ1 was repressed to almost undetectable levels (Supplementary Fig. S4b). Consistent with these data, measurements of enzyme activity showed a much higher DFMO-sensitive ODC activity in *Mgrn1*-null melan-md1 cells compared with melan-a6 controls (Supplementary Fig. S4c). Moreover, *Mgrn1*-KO cells also showed a significant increase in the levels of putrescine, the product of ODC-catalyzed decarboxylation of ornithine. Concerning the other major polyamines, the levels of spermidine were similar in both cell types but those of spermine were lower in melan-md1 cells (Supplementary Fig. S4d). Accordingly, the combined concentration of intracellular polyamines was not significantly different in cells expressing or lacking MGRN1. Moreover, inhibition of polyamine biosynthesis with DFMO lowered polyamine contents in melan-a6 cells and blocked cell proliferation but stimulated two–threefold both TYR activity in situ and melanin content (Supplementary Fig. S4e), in agreement with our previous data [62], thus ruling out a major role of polyamine metabolism in increasing intramelanosomal pH.

Therefore, we focused our attention on *Atp6v0d2* and *Mcoln3.* As stated above, *Atp6v0d2* codes for the d2 subunit of V-ATPase responsible for organellar acidification in eukaryotic cells. V-ATPase activity is regulated in vivo through the reversible association of two multisubunit modules, the V_1_ domain with ATPase activity and the V_o_ domain responsible for proton translocation [63], with the d2 protein partially bridging these domains. Conceivably, overexpression of a subunit located at the interface between the two domains and contacting both of them might interfere with the correct assembly of the complex, much in the same way as overexpression of a scaffold may attenuate its own function [64]. We validated differential expression of *Atp6v0d2* as a function of *Mgrn1* expression levels by qPCR. We found strong overexpression of the gene in melan-md1 cells and *Mgrn1*-KO clones derived from melan-a6 melanocytes or B16 melanoma cells (Supplementary Fig. S5a). Strong and acute downregulation of MGRN1 in melan-a6 cells by means of two independent siRNAs also upregulated *Atp6v0d2* expression (Supplementary Fig. S5b). Immunohistochemical staining of ATP6V0D2 and Western blot analysis confirmed overexpression of the d2 subunit of V-ATPase in melan-md1 compared with melan-a6 cells (Supplementary Fig. S5c). We next measured TYR activity in melan-md1 cells depleted of ATP6V0D2*.* Treatment of melan-md1 cells with 3 different siRNAs specific for *Atp6v0d2* strongly decreased expression of the corresponding mRNA and the ATP6V0D2 protein (Supplementary Fig. S5d) but had a small stimulatory effect, if any, on TYR activity (Supplementary Fig. S5e). Therefore, expression of *Atp6v0d2* was modulated by MGRN1, but was inversely related with TYR enzymatic activity. Accordingly, TYR stimulation in MGRN1-depleted cells could not be accounted for by *Atp6v02d* overexpression in these cells.

**Fig. 5:**
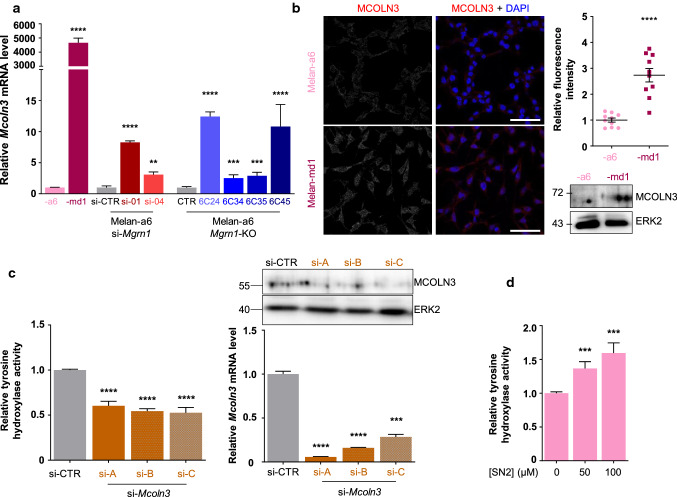
Induction of MCOLN3 expression upon downregulation of MGRN1. **a** RT-PCR analysis of *Mcoln3* expression in melan-a6 and md1 melanocytes, *Mgrn1*-KO melanocytes derived from melan-a6 cells by CRISPR-Cas9 (clones 6C24, 6c34, 6c36 and 6c45) and in melan-a6 cells treated with *Mgrn1*-directed siRNAs 01 and 04. In all cases, relative *Mcoln3* mRNA levels in MGRN1-deprived cells respect to the corresponding controls are shown (*n* ≥ 3). **b** Expression of MCOLN3 protein in melan-a6 or -md1 melanocytes. Confocal microscopy images of cells immunostained for endogenous MCOLN3 (left), histogram corresponding to the quantification of the fluorescence intensity of MCOLN3 staining normalized by number of cells (middle, *n* = 10), and representative immunoblots for endogenous MCOLN3 expression. ERK2 was used as loading control. **c** Effect of downregulation of MCOLN3 on TYR activity of melan-md1 cells. Melan-md1 melanocytes were treated with 3 different *Mcoln3*-directed siRNAs, then tyrosine hydroxylase activity was measured in vivo. The bar graph on the left shows the residual TYR activity in MCOLN3-depleted cells (si-A, -B and -C), relative to controls treated with a scrambled siRNA (siCTR). On the right, the upper Western blot shows the abundance of the MCOLN3 protein in cells treated with a control siRNA (siCTR) or with *Mcoln3*-specific siRNAs, with ERK2 as a control for comparable loading. The lower bar graph depicts the residual *Mcoln3* mRNA levels in these cells as estimated by RT-PCR. **d** Tyrosine hydroxylase activity in live melan-a6 cells treated with the indicated concentrations of the MCOLN3 channel agonist SN2 (*n* ≥ 10).

*Mcoln3* is a coat color gene mapping to the mouse *varitint-waddler* (*Va*) locus, whose mutations cause a phenotype characterized by patches of normal, diluted and white hair [65]. *Mcoln3* codes for mucolipin3, a member of the transient receptor potential channel family also termed TRPML3 which mainly conducts Ca^2+^ ions. Importantly, overexpression of *Mcoln3* has been reported to increase the pH of lysosomes in ARPE cells [66]. We confirmed by qPCR that expression of *Mcoln3* was higher in *Mgrn1*-null or -repressed cells (Fig. 5a), resulting in increased accumulation of the protein as shown by immunostaining or Western blot (Fig. 5b). To test the effects of changes in *Mcoln3* expression on TYR activity, we treated melan-md1 cells with three *Mcoln3*-directed siRNAs. Downregulation of *Mcoln3* expression leading to decreased abundance of MCOLN3 protein consistently and significantly inhibited TYR (Fig. 5c). Moreover, treatment of melan-a6 cells with the specific agonist of the calcium channel SN2 significantly increased TYR activity in a dose-dependent manner (Fig. 5d). Taken together, these data highlighted a positive correlation of *Mcoln3* expression and TYR activity.

**Fig. 6:**
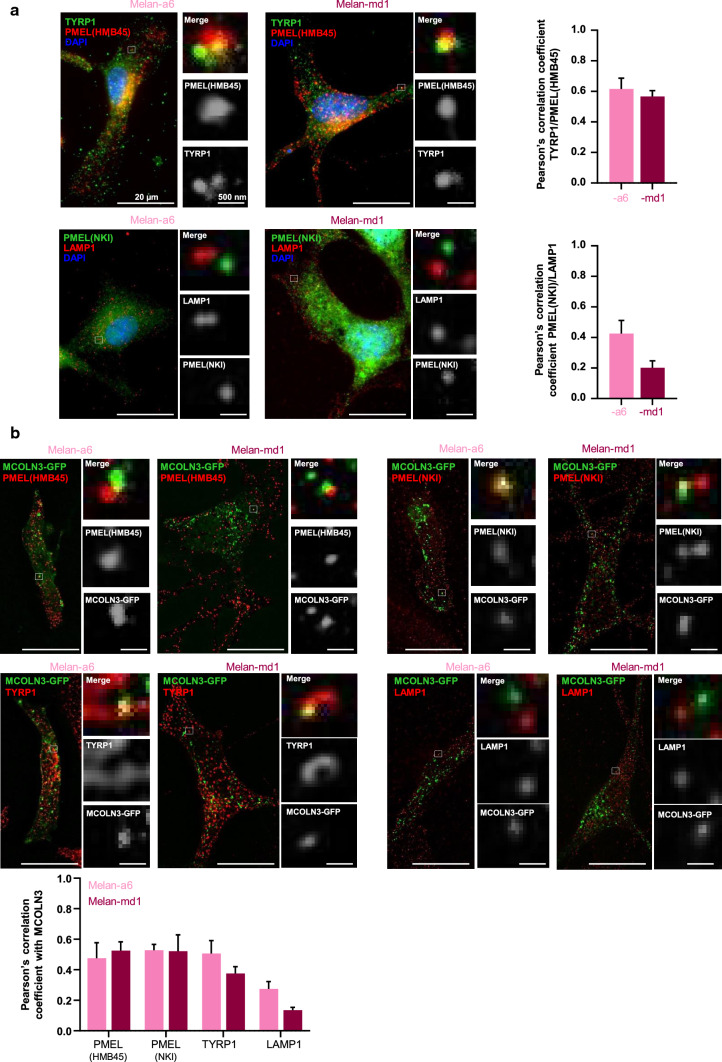
Subcellular localization of MCOLN3 in melanocytes. Confocal microscopy images of melan-a6 and -md1 cells. **a** Images of non-transfected cells labeled for endogenous TYRP1, HMB45 and NKI. The endogenous level of LAMP1 was used as negative control; **b** Cells transfected with an MCOLN3-GFP construct and stained for TYRP1, HMB45, NKI and LAMP1. Pearson’s correlation coefficient between two channels was quantified using ImageJ Fiji from one frame of the cell acquisition.

A number of studies showed the association of MCOLN3 with endosomal compartments (reviewed in Cheng et al. 2010 [60]) but whether this protein is present in melanosomes remains to be investigated. Therefore, we explored the subcellular localization of MCOLN3 in melanocytes. Melan-a6 cells were transfected with an expression vector encoding a GFP-MCOLN3 chimera. Cells were labeled with antibodies directed against two melanosomal proteins, TYRP1 and PMEL, the lysosomal protein LAMP1 and a trans-Golgi component (TGN38). For PMEL, two commercial antibodies, NKI and HMB45 were employed. As positive and negative controls for co-localization, melanocytes were labeled for TYRP1 and PMEL or PMEL and LAMP1, respectively, and the extent of co-localization was estimated using Pearson’s correlation coefficients (Fig. 6a). The GFP-MCOLN3 signal showed a punctate pattern compatible with location to intracellular vesicles. This pattern was similar in melan-a6 and melan-md1 cells, suggesting that MGRN1 expression did not interfere with MCOLN3 intracellular distribution [65, 66] (Fig. 6b). MCOLN3 significantly co-localized with TYRP1 or PMEL, but it did not show a comparable co-localization with LAMP1 (Fig. 6b) or TGN38 (not shown), thus supporting the specificity of the assay. Of note, lack of detectable co-localization with LAMP1 indicated little if any association of MCOLN3 with lysosomes in melanocytes.

**Fig. 7:**
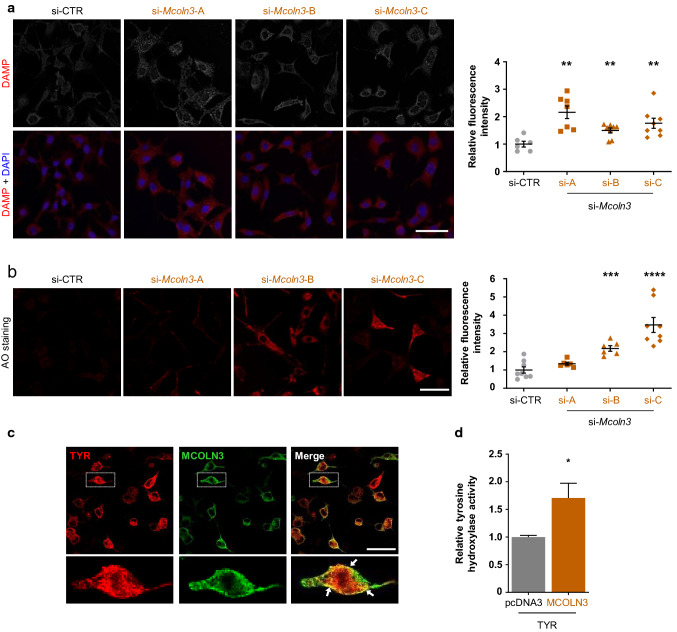
Regulation of the pH of acidic organelles in melanocytic cells by MCOLN3. **a** Organelle acidification in melan-md1 cells upon *Mcoln3* silencing. *Mcoln3* expression was silenced in melan-md1 cells by treatment with 3 different specific siRNAs, as in Fig. 5 panel. DAMP-stained cells were observed with a confocal microscope. The graph on the right shows the quantification of DAMP fluorescence in the confocal microscopy images. Data are normalized to the fluoresce intensity of cells treated with a control scrambled siRNA (siCTR). **b** Same as in panel A, except that cells were stained with AO. **c** Co-localization of MCOLN3 and TYR upon forced expression in HEK293 cells. HEK293 cells were transfected to express a HA epitope-labeled MCOLN3 and Flag-labeled TYR. Both proteins were immunostained and cells observed in a confocal microscope. Strong co-localization of both proteins was detected. **d** Increased TYR activity in cells co-transfected with TYR and MCOLN3 expression constructs. HEK293 cells were transfected with a TYR expression construct and empty vector (pcDNA) or an MCOLN3 construct, as indicated. The tyrosine hydroxylase activity in live cells was measured and data were normalized with the activity measured in cells expressing TYR alone. Scale bar: 50 µm.

Taken together, the data presented above showed that (i) MCOLN3 was upregulated following knockdown of *Mgrn1*, (ii) MCOLN3 could locate to melanosomes in melanocytes and (iii) pharmacological activation of MCOLN3 increased TYR activity whereas repression of its expression with siRNA inhibited the rate-limiting melanogenic step. Moreover, previous reports indicated that MCOLN3 contributes to the control of the pH of organelles within the endosomal pathway [66–69]. Thus, we hypothesized that MCOLN3-dependent neutralization of the melanosomal pH could be partially responsible for the activation of melanogenesis in *Mgrn1*-null melanocytes. To test this hypothesis, we first confirmed a relationship between MCOLN3 expression and intramelanosomal pH. MCOLN3 expression was downregulated by treating melan-md1 cells with the same siRNAs employed above. After 72 h, the cells were stained with DAMP (Fig. 7a) or AO (Fig. 7b). For the different siRNAs tested, fluorescence intensity was significantly higher for both probes, consistent with acidification of intracellular compartments including melanosomes upon downregulation of MCOLN3 expression. We also co-transfected HEK293 cells with HA-labeled MCOLN3 and Flag-tagged TYR. Strong apposition of both proteins was easily detected in confocal microscopy images (Fig. 7c). Importantly, TYR activity was higher in live cells coexpressing the enzyme and MCOLN3 compared with cells transfected with TYR alone (Fig. 7d), in keeping with neutralization of lysosomal pH.

## Discussion

One of the most conspicuous consequences of the loss-of-function *Mahoganoid* mutation abrogating *Mgrn1* expression in mice is coat color darkening and promotion of eumelanin synthesis [70]. MGRN1, a protein involved in the regulation of the switch from eumelanogenesis to pheomelanogenesis [32, 71], is known to inhibit signaling downstream of MC1R [31, 41]. However, the stimulation of melanogenesis in cultured *Mgrn1*-null mouse melanocytes appears to be a cell-autonomous process independent on activation of MC1R by exogenous agonists [32, 33]. Consistent with this, *mahoganoid* melan-md1 and genetically matched melan-a6 melanocytes express comparable levels of the MITF transcription factor, a key downstream target of MC1R and a major transcriptional regulator of the *Tyr* gene [33]. In keeping with these results, here we showed that *Mgrn1* knockdown stimulated TYR activity in live cells, as well as melanin pigment production, without significant changes in the expression or post-translational processing of TYR. Therefore, lack of MGRN1 increased TYR-specific activity rather than abundance.

Accumulating evidence show that modulation of the intraluminal pH of melanosomes is a major control mechanism in melanin biosynthesis. The melanosomal pH acts at different levels to regulate the rate of eumelanogenesis. It likely affects the rate of maturation and/or trafficking of melanosomal proteins, as proteins known to modulate the melanosomal pH such as OCA2 facilitate TYR processing and transport [72–74]. It also has a strong effect on the specific activity of the rate-limiting TYR, which is higher at neutral pH compared with the acidic pH typical of late endosomes, lysosomes and premelanosomes [75–78]. Moreover, the luminal pH is a key determinant of the reactivity, fate and speed of polymerization of melanogenic intermediates. Notably, protonation of the amino group in the side chain of DOPAquinone is a major determinant of its reactivity as a nucleophilic group. Thus, intramolecular cyclization of DOPAquinone, and hence eumelanogenesis as opposed to pheomelanogenesis, are favored in less acidic media [79]. In agreement, weakly acidic pH values typical of the lumen of late endosomes and premelanosomes greatly suppress eumelanin production [80] while promoting pheomelanogenesis [81].

Using a variety of pH-sensitive fluorescent probes, we found that downregulation of MGRN1 expression significantly increased the pH of acidic organelles, thus accounting for the high specific activity of TYR in the melanosomes of MGRN1-deficient melanocytes and melanoma cells. Therefore, MGRN1 most likely promoted an acidic melanosomal pH, since its loss had a neutralizing effect. The resulting activation of TYR would augment the rate of melanin production and deposition within melanosomes, thus accelerating melanosome maturation. This would account for the greater percentage of melanized stage III and IV melanosomes as compared with poorly pigmented stage I and II melanosomes in MGRN1-deficient cells observed in this study. Moreover, both the higher TYR activity and the expected changes in the reactivity of melanogenic intermediates at near-neutral pH are consistent with previous observations that lack of MGRN1 induces eumelanogenesis while abrogating pheomelanogenesis [16, 82].

The regulation of melanosomal pH is extremely complex and the underlying molecular mechanisms remain largely unknown, in spite of recent progress. Many of the proteins that contribute to setting the melanosomal pH have been identified, most often with the help of mouse genetics. These include OCA2 (also known as P) [83], SLC45A2 (also named MATP or AIM1) [84] and TPC2 [85, 86]. However, this list is likely incomplete and their relative contribution, as well as their precise mechanism of action, remain unknown. Nevertheless, a general picture is emerging, where the acidic pH of premelanosomes, LROs derived from endosomal vesicles, is established by the active intake of protons from the cytosol mediated by a V-type ATPase. During melanosome maturation, the melanosomal pH is modulated owing to the activity of membrane transport proteins such as SLC45A2, OCA2/P, TPC2, the Cl^−^/H^+^ exchanger CLC-7 mutated in a subset of albinism patients [87] and possibly other still unidentified Ca^2+^ channels. The effect of these proteins on melanosomal pH may be indirect and independent of their ability to act as H^+^ pores, as TPC2 and OCA2/P are best known as Na^+^/Ca^2+^ and chloride channels, respectively (reviewed in Wiriyasermkul et al. 2020 [88]). Importantly, the acidic pH of premelanosomes favors the assembly of the PMEL fibrils that form the internal protein network over which melanins are progressively deposited during melanosome maturation [89–91], whereas the higher pH in stage III and IV melanosomes promotes the formation of melanin pigments by activating TYR [75–78]. Accordingly, it appears that during melanosome maturation an increase of the intraluminal pH from an acidic value to a near-neutral situation takes place in the transition from stage II to stage III, resulting in activation of melanin synthesis, with preference for eumelanogenesis as opposed to pheomelanogenesis. This suggests that not only the final setpoint of the pH in melanogenically active melanosomes, but also the rate at which this pH is achieved, are key factors in the regulation of the quantity and quality of the pigment produced. Of note, the efficiency and rate of neutralization of the melanosomal lumen are dependent on genetic factors [77] and may be regulated by extracellular cues, most notably stimulation of MC1R by its natural agonist, αMSH [92]. It is also worth noting that a cAMP-independent signaling pathway acting via MC1R and MGRN1 has been proposed to mediate biological effects of Agouti signaling protein (ASIP), a protein that regulates switching between eumelanin and pheomelanin synthesis in melanocytes [32].

In keeping with a role of MGRN1 as a new regulator of melanosomal pH, *Mgrn1* knockdown significantly altered the expression of several potential pH regulatory genes. Notably, *Atp6v0d2* and *Mcoln3* were significantly upregulated. Therefore, we analyzed their possible involvement in the pigmentation changes following depletion of MGRN1. Concerning *Atp6v0d2*, its siRNA-mediated downregulation increased TYR activity in melan-a6 cells. Thus, the activation of the rate-limiting melanogenic enzyme in MGRN1-deficient cells was most likely independent on the observed increase in *Atp6v02d* expression following depletion of MGRN1. It has been shown that V-ATPase is mostly found in acidic premelanosomes, but not in mature and highly pigmented melanosomes [93]. Moreover, several studies using zebrafish mutants in V-ATPase subunits strongly suggested that V-ATPase expression is required for melanosome biogenesis and/or to sustain normal melanosome morphology and stability [94, 95]. Conceivably, the overexpression of *Atp6v0d2* and other V-ATPase subunits in MGRN1-depleted cells might, therefore, be related with the increased number of melanosomes in these cells rather with the high specific activity of TYR in these melanosomes.

Of interest, the effect of MGRN1 on melanosomal pH appeared largely dependent on changes in the expression of MCOLN3. This integral membrane protein belongs to the mucolipin family of transient receptor potential channels, whose best-known member is MCOLN1. *Mcoln1* is mutated in type IV mucolipidosis, a lysosomal storage disorder, and is believed to regulate late endocytic trafficking and lysosomal acidification among other functions [96–99]. In keeping with an acidifying action, both a disease-causing loss-of-function mutation in *Mcoln1* or its downregulation with specific siRNA led to neutralization of the lysosomal pH in MLIV and HeLa cells [99]. *Mcoln3* is known as a coat color gene mutated in the *varitint-waddler* mouse [65], with certain mutations causing complete absence of coat melanin among other phenotypes. MCOLN3 behaves as a ionic channel allowing movement of cations from the organellar lumen to the cytosol. Although it shows a preference for monovalent cations, it is also permeable to Ca^2+^ but not H^+^ [60]. Work by Martina et al. showed that in ARPE19 retinal epithelial cells, MCOLN3 localizes to early and late endosomes and is likely involved in protein trafficking along the endosomal pathway [66]. Using pH-sensitive fluorescent probes, it was also shown that the average pH of endosomes was higher in cells overexpressing *Mcoln3*, in agreement with a role of MCOLN3 in neutralization of endosomal compartments [66]. However, it has also been reported that inhibition of MCOLN3 reduced endosomal acidification [67]. Accordingly, the role of this transporter in the regulation of the pH is complex and maybe even context-specific.

Based on co-localization with the melanosomal protein TYRP1, we have shown here that MCOLN3 is present in melanosomes of mouse melanocytes. Moreover, downregulation of MCOLN3 expression decreased the melanosomal pH as demonstrated by higher intensity of DAMP and AO staining. In heterologous HEK293 cells lacking melanosomes, MCOLN3 most likely located to lysosomes where it might act to increase the luminal pH as suggested by stimulation of ectopically expressed TYR. The precise mechanism accounting for the neutralizing action of MCOLN3 remains undetermined. However, it seems likely that MCOLN3-mediated neutralization of the pH of LROs is mediated by movement of Ca^2+^ ions. The importance of Ca^2+^ transport for the control of the pH is underlined by the less efficient acidification of endosomes in cells grown in low Ca^2+^ media [67]. Ca^2+^ release from endosomes may regulate the activity of ion channels important for charge compensation, to ensure the correct equilibrium of charges determining the final pH of the organelle. Moreover, soluble adenylyl cyclase, an enzyme recently shown to participate in the acute control of the melanosomal pH by stimulating a non-canonical cAMP-dependent cascade is regulated by Ca^2+^ ions [56].

It remains to be determined how repression of MGRN1 resulted in accumulation of MCOLN3. The simple explanation that MCOLN3 might be a substrate of the E3 ligase activity of MGRN1, so that downregulation of MGRN1 would augment the stability of MCOLN3, is not satisfactory, since we have observed increased *Mcoln3* mRNA levels in MGRN1-deprived cells, thus pointing to a transcriptional mechanism. In this regard, it should be noted that melanocytic cells express several MGRN1 isoforms, and that at least two of them contain nuclear localization signals and locate to nuclei in the presence of MC1R [31]. Accordingly, MGRN1 may modulate the expression of genes such as *Mcoln3* by still uncharacterized mechanisms. It will be interesting to determine if some of these genes are also αMSH- and/or ASIP-responsive and if the interaction of MC1R and MGRN1 can modulate the effect of MGRN1 on melanosomal pH.

## Supplementary Information

Below is the link to the electronic supplementary material.

Supplementary file1 (PPTX 11529 KB) Suppl. Fig. S1 Ultrastructural analysis of MGRN1-depleted MNT-1 human melanoma cells. MNT-1 cells were treated with control (siCTR) or two different MGRN1-specific siRNAs (siMGRN1-03 and -04). a 60-nm electron micrographs showing melanosomes in different maturation stages indicated by I to IV. b Distribution of melanosomal stages in the cellular soma of MNT-1 cells treated with MGRN1-specific siRNA (n≥10). N, nucleus. c Relative mRNA levels estimated by qPCR following treatment of MNT-1 cells with siCTR or siMGRN1-03 and -04 (n=3). Scale bar: 2 µm. Suppl. Fig. S2. a Glycosylation status of TYR in melan-a6 and -md1 cells. Control extracts and extracts treated with endoglycosidase H (EndoH) or peptide-N-glycosidase F (PNGaseF) were analyzed by Western blot. αPEP7 was used for the detection of TYR. A representative immunoblots is shown, where the mature EndoH-resistant TYR band is highlighted by an arrow. ERK2 was used as loading control. b Relative tyrosine uptake by melan-a6 and -md1 melanocytes (n=6), measured as radioactive tyrosine content in cells in the presence or absence of the TYR inhibitor PTU. c Tyrosine hydroxylase activity of cell-free extracts from melan-a6 and -md1 cells measured by the conventional in vitro radiometric assay performed in phosphate buffer at different pH values (n=3). Results are normalized to the activity at pH 5.0. d Tyrosine hydroxylase activity of control and NH4Cl-treated melan-a6 and -md1 melanocytes measured in live cells (n≥4). In each case, the results are shown after normalization to the enzymatic activity measured in the absence of NH4Cl. e Bright field micrographs and cell pellets of cells treated as in panel D. f Intracellular melanin content of control and NH4Cl-treated melan-a6 and -md1 melanocytes (n≥4). Data are normalized by protein content and referred to control. Suppl. Fig. S3 Analysis of the pH of acidic organelles in Mgrn1-null mouse melanocytic cells. a Confocal images of melan-a6 and -md1 melanocytes stained for DAMP-DNP using two different fluorescent probes, Alexa-647 (red) and Alexa-488 (green). In both cases, nuclei were stained with DAPI and merged images of DAMP and DAPI staining are shown on the right. b Histogram showing the quantification of the fluorescence intensity of DAMP staining shown in (D). n≥8 and data normalized to control. c Confocal microscopy images of CRISPR/Cas9 Mgrn1-KO clones of melan-a6 melanocytes labelled for 20 min with AO. CTR stands for control, 6C34 and 6C45 are two independent Mgrn1-KO clones. The quantification of the fluorescence intensity relative to control (n=12) is shown on the right graph. d Same as in panel A, except that two independent clones derived from B16 mouse melanoma cells (clones 5C5 and 6C26) were analyzed. The quantification of the fluorescence intensity of 12 images is shown on the right. Suppl. Fig. S4 Modulation of polyamine metabolism in Mgrn1-null cells. a Heat map of the top 50 most up- or down-regulated genes in melan-md1 cells compared with melan-a6 cells. Genes known to be involved in the regulation of the pH of subcellular compartments are highlighted with arrows. b Relative expression of key genes of the polyamine biosynthetic pathway in melan-md1 and melan-a6 cells. mRNA levels for the indicated genes were analyzed by qPCR. c ODC activity in control and DFMO-treated mouse melanocytes. Values are given after normalization to the activity of melan-a6 cells measured in the absence of DFMO. d Intracellular polyamine level in melan-md1 and -a6 melanocytes. e Tyrosine hydroxylase activity in live melan-a6 and -md1 cells, with or without pretreatment with the ODC inhibitor DFMO. Suppl. Fig. S5 ATP6V0D2 expression in control and MGRN1-deprived melanocytes and effects on tyrosine hydroxylase activity. a Relative expression of Atp6v0d2 in melan-a6 and melan-md1 melanocytes (n=3, left) and CRISPR/Cas9 Mgrn1-KO clones derived from melan-a6 melanocytes and B16F10 mouse melanoma cells (n≥3, right). The abundance of each mRNA was estimated by qPCR, and data are expressed as relative expression compared with the corresponding controls. b Relative expression of Atp6v0d2 in melan-a6 cells treated with control siRNA (siCTR) or Mgrn1-directed siRNAs (si-01 or -04, n≥3). c Confocal images of melan-a6 and melan-md1 cells immunostained for endogenous ATP6V0D2. The histogram shows the relative fluorescence intensity normalized to melan-a6 cells (n≥6). Also shown is a representative immunoblot of ATP6V0D2 expression in mouse melanocytes. ERK2 was used as loading control. d Efficient Atp6v0d2 repression in melan-md1 cells treated with three different Atp6v0d2-directed siRNAs (si-A, -B and –C), as analyzed by qPCR. The upper Western blot shows the relative expression of ATP6V0D2 protein in cells treated in the same conditions, with ERK2 as loading control. e Tyrosine hydroxylase activity measured in live melan-md1 melanocytes treated with the different Atp6v0d2-directed siRNAs (n=4). Data were normalized for total protein content and were expressed as relative activity respect to melanocytes treated with a scrambled control siRNA (siCTR).

## Data Availability

The datasets generated during and/or analyzed during this study are available from the corresponding author on reasonable request. Supporting data can be found.
