# Supplementary material for: Mahogunin Ring Finger 1 regulates pigmentation by controlling the pH of melanosomes in melanocytes and melanoma cells
Source: Cell Mol Life Sci. 2021 Dec 18;79(1):47. doi: 10.1007/s00018-021-04053-9 (PMC8738503; doi:10.1007/s00018-021-04053-9)
Supplement: Supplementary file 1 — Supplementary file1 (PPTX 11529 KB) Suppl. Fig. S1 Ultrastructural analysis of MGRN1-depleted MNT-1 human melanoma cells. MNT-1 cells were treated with control (siCTR) or two different MGRN1-specific siRNAs (siMGRN1-03 and -04). a 60-nm electron micrographs showing melanosomes in different maturation stages indicated by I to IV. b Distribution of melanosomal stages in the cellular soma of MNT-1 cells treated with MGRN1-specific siRNA (n≥10). N, nucleus. c Relative mRNA levels estimated by qPCR following treatment of MNT-1 cells with siCTR or siMGRN1-03 and -04 (n=3). Scale bar: 2 µm. Suppl. Fig. S2. a Glycosylation status of TYR in melan-a6 and -md1 cells. Control extracts and extracts treated with endoglycosidase H (EndoH) or peptide-N-glycosidase F (PNGaseF) were analyzed by Western blot. αPEP7 was used for the detection of TYR. A representative immunoblots is shown, where the mature EndoH-resistant TYR band is highlighted by an arrow. ERK2 was used as loading control. b Relative tyrosine uptake by melan-a6 and -md1 melanocytes (n=6), measured as radioactive tyrosine content in cells in the presence or absence of the TYR inhibitor PTU. c Tyrosine hydroxylase activity of cell-free extracts from melan-a6 and -md1 cells measured by the conventional in vitro radiometric assay performed in phosphate buffer at different pH values (n=3). Results are normalized to the activity at pH 5.0. d Tyrosine hydroxylase activity of control and NH4Cl-treated melan-a6 and -md1 melanocytes measured in live cells (n≥4). In each case, the results are shown after normalization to the enzymatic activity measured in the absence of NH4Cl. e Bright field micrographs and cell pellets of cells treated as in panel D. f Intracellular melanin content of control and NH4Cl-treated melan-a6 and -md1 melanocytes (n≥4). Data are normalized by protein content and referred to control. Suppl. Fig. S3 Analysis of the pH of acidic organelles in Mgrn1-null mouse melanocytic cells. a Confocal images of melan [file 18_2021_4053_MOESM1_ESM.pptx]

## Slide 1
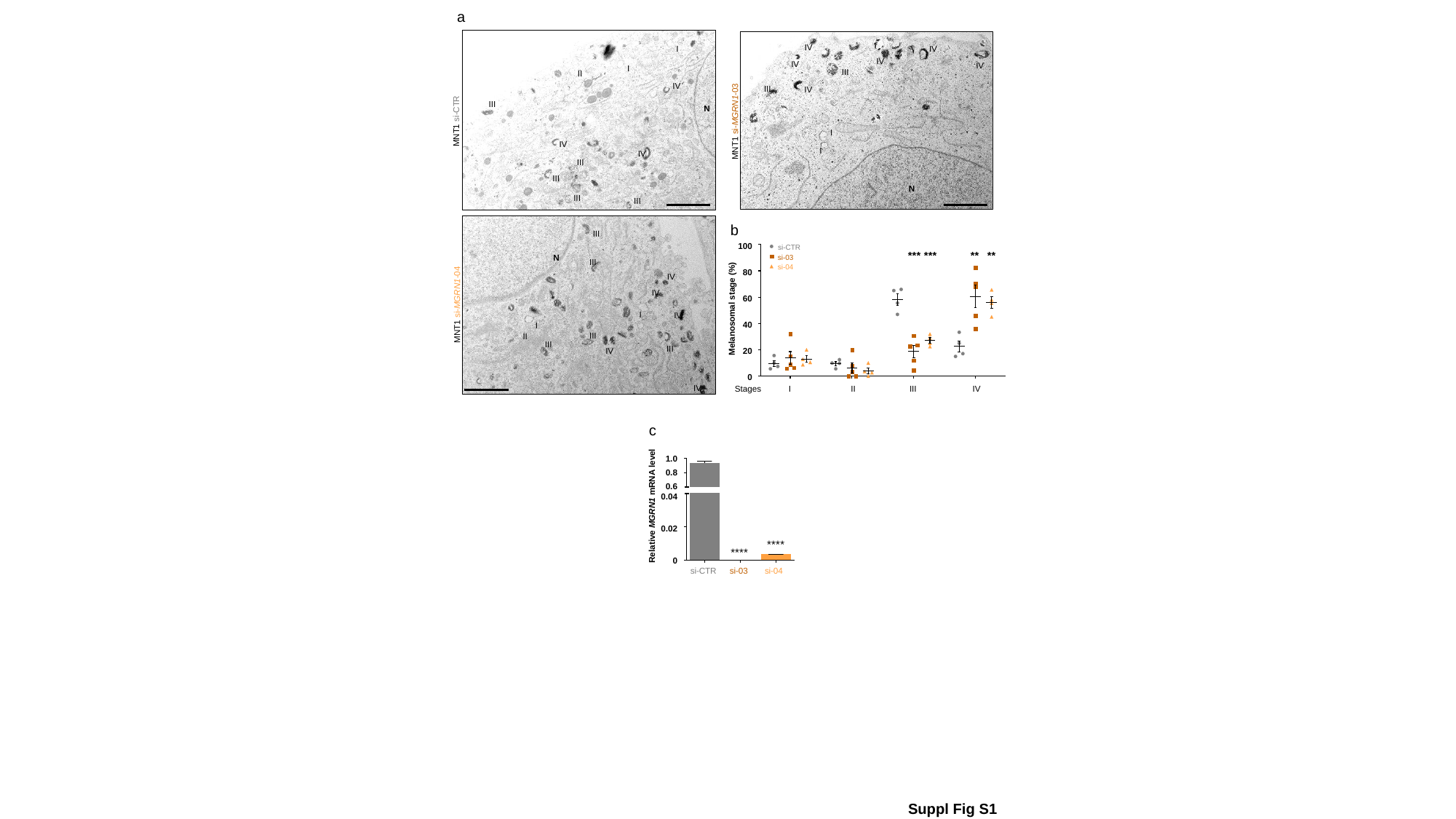

a
I
I
II
IV
III
N
MNT1 si-CTR
IV
IV
III
III
III
III
IV
IV
IV
IV
IV
III
III
IV
MNT1 si-MGRN1-03
I
I
N
b
III
N
III
IV
IV
MNT1 si-MGRN1-04
I
IV
I
III
II
III
III
IV
IV
100
si-CTR
si-03
si-04
80
60
Melanosomal stage (%)
40
20
0
Stages
I
II
III
IV
c
1.0
0.8
0.6
0.04
0.02
0
si-CTR
si-03
si-04
Relative MGRN1 mRNA level
Suppl Fig S1

## Slide 2
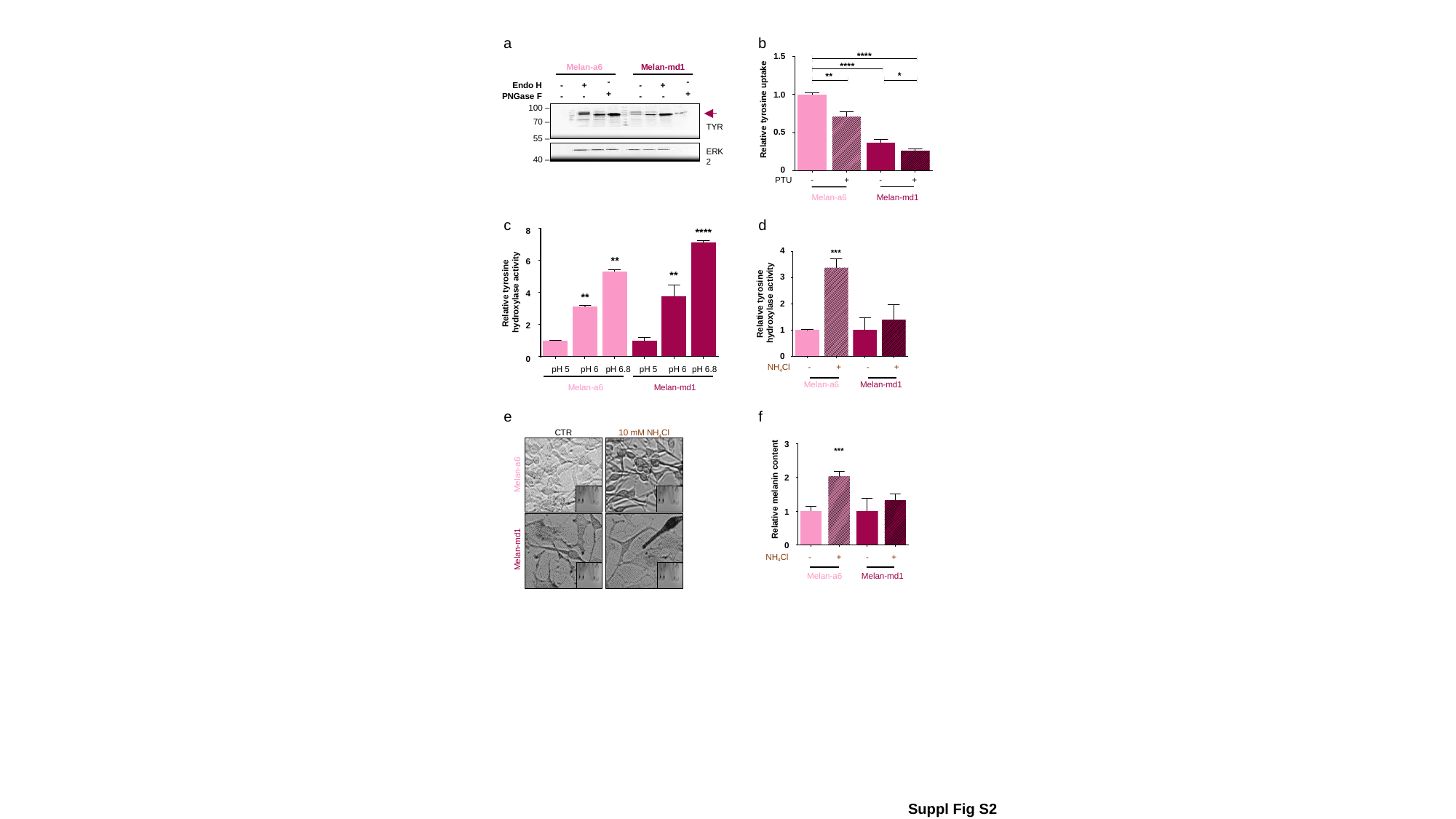

a
b
Relative tyrosine uptake
PTU
-
+
-
+
Melan-a6
Melan-md1
1.5
1.0
0.5
0
Melan-a6
Melan-md1
+
-
+
-
Endo H
-
-
+
-
-
-
+
-
100 –
70 –
55 –
40 –
PNGase F
TYR
ERK2
c
d
Relative tyrosine
hydroxylase activity
pH 5
pH 6
pH 6.8
pH 5
pH 6
pH 6.8
Melan-a6
Melan-md1
8
6
4
2
0
4
Relative tyrosine
hydroxylase activity
NH4Cl
-
+
-
+
Melan-a6
Melan-md1
3
2
1
0
e
f
CTR
10 mM NH4Cl
Melan-a6
Melan-md1
Relative melanin content
NH4Cl
-
+
-
+
Melan-a6
Melan-md1
3
2
1
0
Suppl Fig S2

## Slide 3
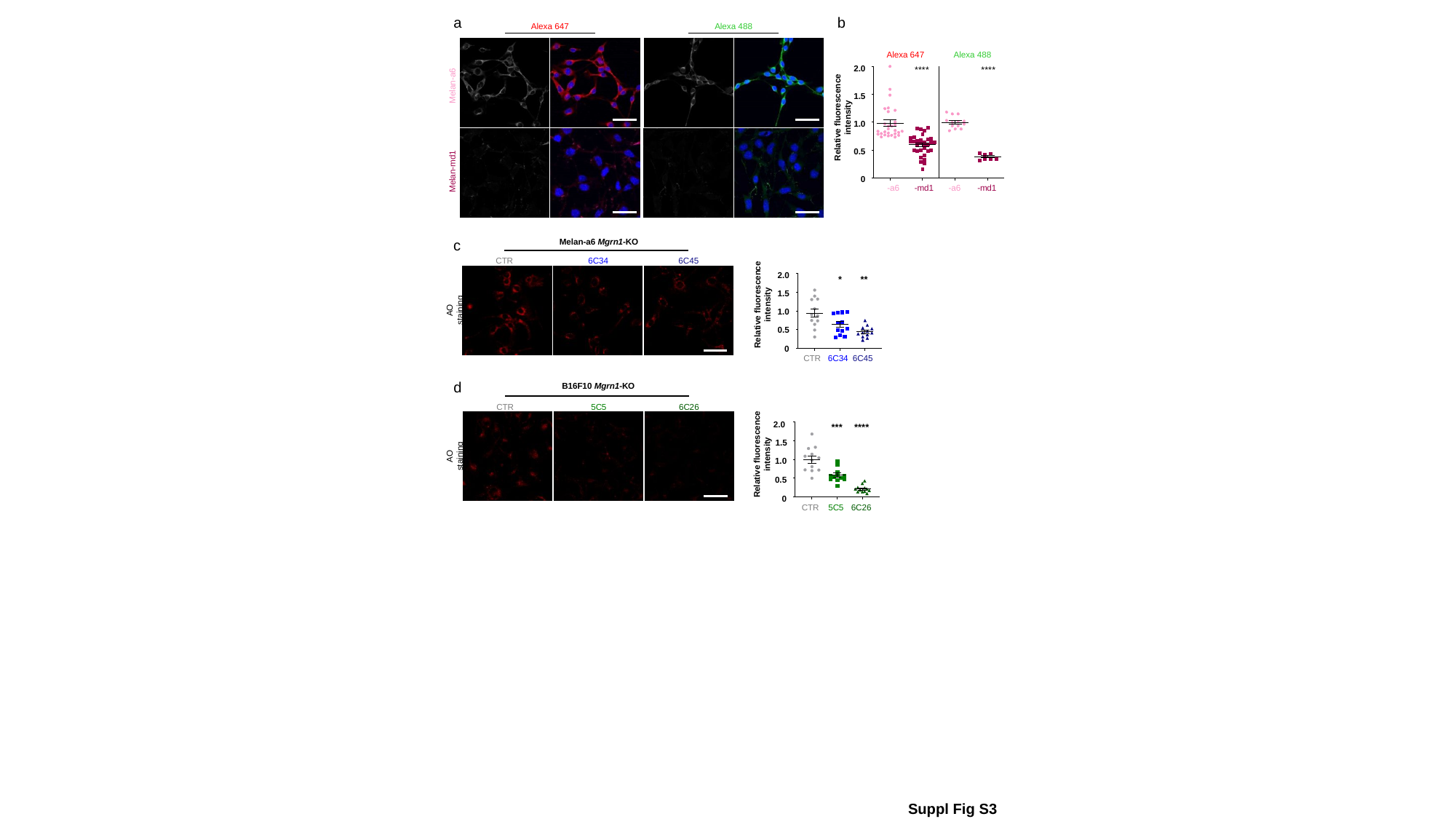

a
b
Alexa 647
Alexa 488
Melan-a6
Melan-md1
Alexa 647
Alexa 488
2.0
1.5
Relative fluorescence intensity
1.0
0.5
0
-a6
-md1
-a6
-md1
c
Melan-a6 Mgrn1-KO
CTR
6C34
6C45
2.0
1.5
Relative fluorescence
intensity
1.0
0.5
0
CTR
6C34
6C45
AO staining
d
B16F10 Mgrn1-KO
CTR
5C5
6C26
2.0
1.5
Relative fluorescence
intensity
1.0
0.5
0
CTR
5C5
6C26
AO staining
Suppl Fig S3

## Slide 4
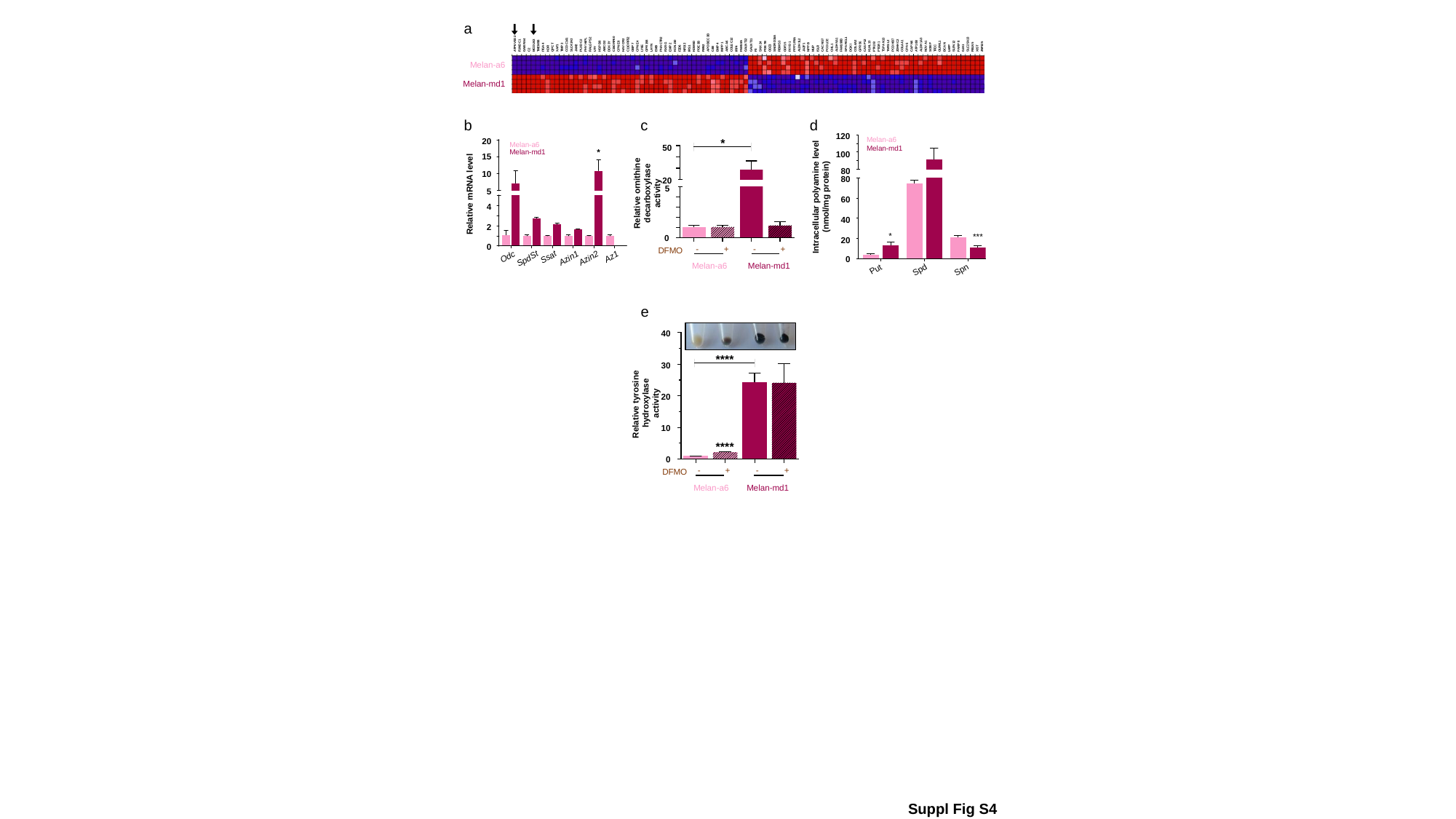

a
Melan-a6
Melan-md1
b
c
d
120
Melan-a6
Melan-md1
100
80
80
Intracellular polyamine level
(nmol/mg protein)
60
40
20
0
Put
Spd
Spn
20
Melan-a6
Melan-md1
15
10
5
Relative mRNA level
4
2
0
Az1
Odc
Ssat
Azin1
Azin2
SpdSt
50
20
Relative ornithine
decarboxylase activity
5
0
-
+
-
DFMO
Melan-a6
Melan-md1
+
e
40
30
Relative tyrosine
hydroxylase activity
20
10
0
+
-
+
-
DFMO
Melan-a6
Melan-md1
Suppl Fig S4

## Slide 5
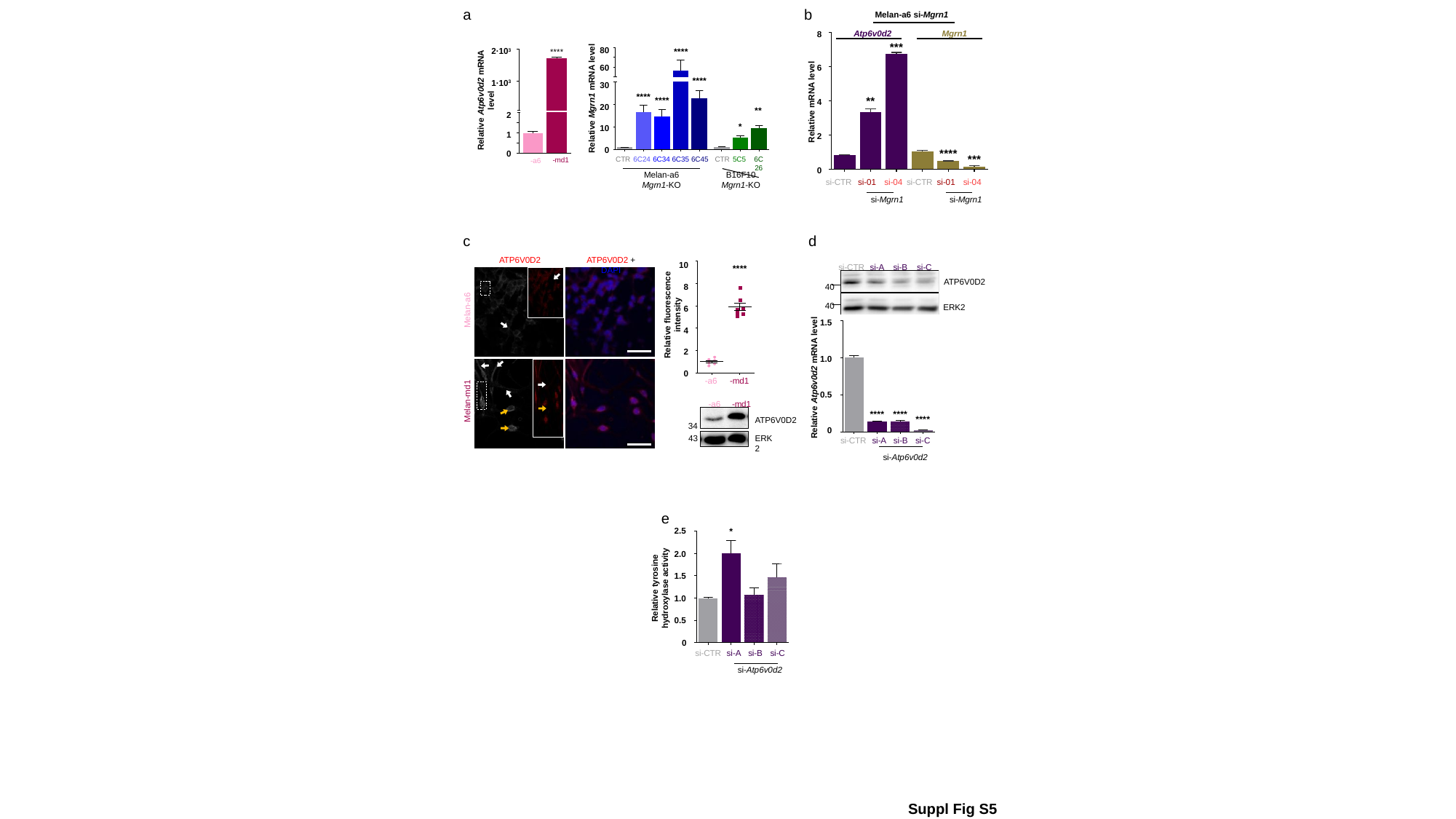

a
b
Melan-a6 si-Mgrn1
Atp6v0d2
Mgrn1
8
6
Relative mRNA level
4
2
0
si-CTR
si-01
si-04
si-CTR
si-01
si-04
si-Mgrn1
si-Mgrn1
-md1
-a6
2·103
1·103
2
1
0
Relative Atp6v0d2 mRNA level
80
60
30
Relative Mgrn1 mRNA level
20
10
0
CTR
6C24
6C34
6C35
6C45
CTR
5C5
6C26
Melan-a6
Mgrn1-KO
B16F10
Mgrn1-KO
c
d
ATP6V0D2
ATP6V0D2 + DAPI
Melan-a6
Melan-md1
-a6
-md1
10
8
6
Relative fluorescence
intensity
4
2
0
si-CTR
si-A
si-B
si-C
ATP6V0D2
40
40
ERK2
1.5
1.0
0.5
0
si-CTR
si-A
si-B
si-C
Relative Atp6v0d2 mRNA level
si-Atp6v0d2
-a6
-md1
ATP6V0D2
34 –
43 –
ERK2
e
2.5
2.0
1.5
Relative tyrosine hydroxylase activity
1.0
0.5
0
si-CTR
si-A
si-B
si-C
si-Atp6v0d2
Suppl Fig S5
